# Photophysical Investigation of Coumarin Solubilization in Polyethylene Glycol Mediated by a 1,2,4-Triazolium Protic Ionic Liquid

**DOI:** 10.1021/acsomega.6c06167

**Published:** 2026-08-14

**Authors:** Saranya Cheriyathennatt, Surya Saravanan, Srinivasan Gokul Raj, Susithra Selvam, Elango Kandasamy

**Affiliations:** † Department of Chemistry, Amrita School of Physical Sciences, 77649 Amrita Vishwa Vidyapeetham, Coimbatore 641112, India; ‡ Functional Materials Laboratory, Amrita School of Engineering, Amrita Vishwa Vidyapeetham, Coimbatore 641112, India; § Department of Physics, 28546 Pondicherry University, Kalapet, Puducherry 605014, India; ∥ Department of Chemistry, Ethiraj College For Women, Ethiraj Salai Egmore, Chennai, Tamil Nadu 600008, India

## Abstract

Polymers are remarkable macromolecules that can be fabricated with ease for the required potential applications, including biotechnology, medicine, engineering, and nanotechnology. Biocompatible and biodegradable polymers are now widely adopted in pharmaceutical formulations, since they offer better solubilization and enhanced bioavailability. One of the biggest challenges to pharmaceutical research is the poor aqueous solubility of hydrophobic drug molecules, which results in limited bioavailability and reduced therapeutic performance. In this work, well-established biocompatible polymer polyethylene glycol (PEG) is employed as the pharmaceutical excipient for the hydrophobic drug coumarin (COU). Along with PEG, a synthesized room-temperature triazolium-based protic ionic liquid (PIL) was incorporated as an additive, and their synergetic effect on COU solubilization was studied by exploring the photophysical properties of COU using steady-state and time-resolved fluorescence techniques. The experiments reveal that incorporation of COU into the PEG/IL system significantly alters the emission intensity, spectral position, and excited-state lifetime, indicating strong nanoenvironmental modulation. Quenching studies indicate the controlled accessibility of COU, suggesting that it is partially confined within PEG rather than entirely encapsulated. A 1,2,4-triazolium-based PIL/polymer platform enhances COU solubilization, where the polymer provides a confined environment for improved solubility and stability and the PIL enables fine control over local polarity, structural organization, and molecular accessibility. Overall, these results demonstrate that PEG/IL systems provide a versatile and reliable platform for enhancing the solubility, stability, and photophysical properties of hydrophobic drug molecules, which underscores their promise for advanced pharmaceutical formulations and sensing applications.

## Graphical abstract

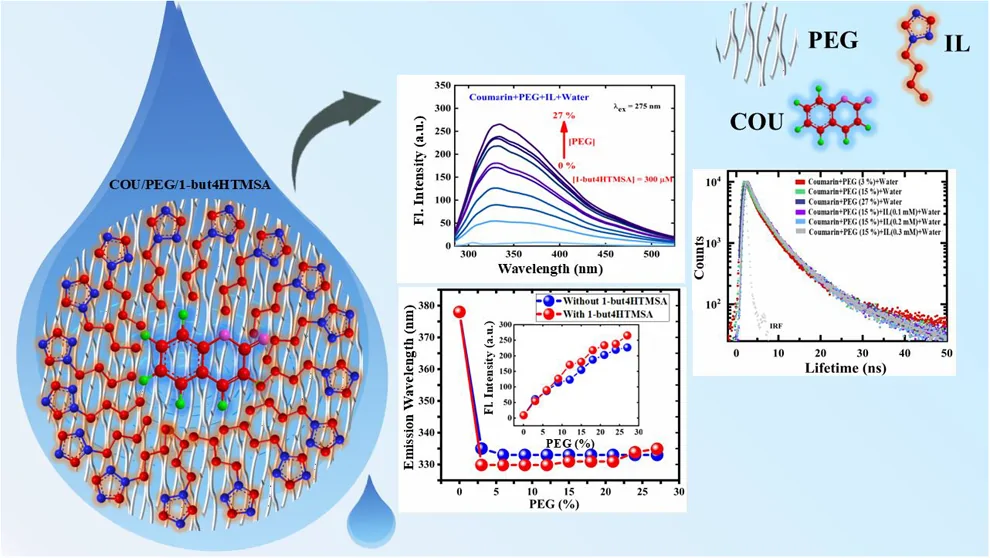

## 1 Introduction

Biocompatible polymers are drawing a lot of interest in pharmaceutical and biomedical applications because of their adaptability, safety, and adjustable physicochemical characteristics.^1,2^ These polymers are made to support or enhance physiological functions without interfering with normal biological activity. Thus, biocompatible polymers have become widely used in controlled drug delivery systems, scaffolds, implants, artificial grafts, wound dressings, bone fillers, and tissue engineering.^2–4^

Even with great advancements in drug discovery, the development of pharmaceutical formulations is still greatly hampered by the poor aqueous solubility of many therapeutic compounds. Many recently developed drug molecules have low solubilities in biological fluids due to their hydrophobic nature. Since aqueous solubility and permeability across biological membranes are the primary factors governing drug bioavailability, inadequate solubilization directly leads to poor absorption and decreased therapeutic efficacy.^5^ Significantly, solubility enhancement is a necessary precondition for effective drug delivery, since only the solubilized fraction of a drug is available for membrane transport and subsequent pharmacological activity. Biocompatible polymers have played a long-standing role in drug delivery systems; initially, they were used for standard purposes such as bulking agents, binders, or swelling matrices that help the drug release when it comes into contact with gastrointestinal media.^6^ In controlled drug delivery, polymers serve as encapsulating carriers and excipients that protect active pharmaceutical ingredients, extend shelf life, and regulate drug release profiles.^7^ Properly formulated polymeric excipients can be made to interact well with particular drugs, thereby boosting formulation quality, safety, and performance.^8,9^

Among polymeric excipients, polyethylene glycol (PEG) has emerged as one of the most popular biodegradable polymers due to its superior hydrophilicity, excellent biocompatibility, and safety profile.^10–12^ Hydrophobic molecules can be accommodated in a confined, hydrated microenvironment by PEG, which increases their apparent solubility and stability.^13,14^ PEG has been extensively employed in pharmaceutical formulations to improve the performance of therapeutic agents through various formulation strategies.^8,9^ PEGs are utilized in cosmetic and personal care products as humectants, emulsifiers, and penetration enhancers in addition to drug delivery.^14–16^ Given its versatility, safety, and multifunctional performance, PEG remains an essential polymer in modern formulation science and therapeutic design.^17,18^

However, polymer-based systems by themselves might not always offer enough control over the local nanoenvironment, including polarity, rigidity, and molecular accessibility, which strongly influence drug behavior at the molecular level. To address these drawbacks, the incorporation of functional additives that can synergistically interact with polymeric matrices has gained increasing attention. In this context, ionic liquids (ILs) have garnered attention due to their unique solvation characteristics, tunable polarity and hydrophobicity, adjustable viscosity, and ability to facilitate enhanced drug loading and bioavailability and ability to modulate intermolecular interactions through rational cation–anion design, and high thermal and chemical stability.^19,20^ Among the different IL families, imidazolium-based ILs have been frequently studied for their potential in biomedical applications.^21,22^ However, according to recent findings, triazolium-based ILs demonstrate greater chemical and thermal stability compared with imidazolium-based ILs, making them promising alternatives.^23^ They also possess high polarity.^24^ In recent years, studies exploring their applications in diverse systems have begun.^25–29^ As a result, 1,2,4-triazolium-based ILs are anticipated to be investigated more thoroughly in order to modulate the physicochemical and biopharmaceutical attributes of hydrophobic drug molecules, thereby enhancing their formulation stability.

Sensitive molecular-level probes are necessary to comprehend the nature of such polymer/additive/drug interactions. Since the photophysical properties of fluorescent molecules are extremely sensitive to changes in their surrounding environment, fluorescence spectroscopy is a powerful tool for this purpose.^30,31^ Coumarin (COU), a hydrophobic fluorophore, is used as a model drug because of its sensitivity to surrounding factors such as polarity, confinement, and molecular interactions. Coumarins are heterocyclic compounds that are members of the benzopyrone family and are defined by the fusion of a pyrone ring with a benzene ring.^32^ They are a diverse and extensively dispersed class of natural products that are found throughout the plant kingdom.^33^ COUs exhibit a broad spectrum of biological activities, including antibacterial, antioxidant, anti-inflammatory, and anticancer effects; oncology research is paying special attention to their anticancer potential.^33,34^ Their diverse physiological, bacteriostatic, and antitumor properties make coumarins versatile scaffolds for structural modification and the development of novel therapeutic agents.^33,34^ Beyond their biological relevance, coumarins also find use in photonics and light-based technologies due to their favorable optical properties.^35^ The basic COU structure displays negligible blue fluorescence, characterized by a negligible quantum yield in water.^33^ Numerous studies have focused on the synthesis and photophysical characterization of substituted COU derivatives; the parent COU molecule has received comparatively limited attention, particularly in aqueous environments, owing to its poor solubility, limited stability, and low bioavailability.^33,36–38^ However, upon binding to specific macromolecular environments, the fluorescence quantum yield increases significantly.

In this study, COU is incorporated into a polyethylene glycol (PEG) matrix, while the 1,2,4-triazolium-based PIL, 1-but4HTMSA, is introduced as an additive to probe its role in aqueous solubilization and stabilization. The synergistic influence of PEG and 1-but4HTMSA on the photophysical behavior of COU is systematically investigated using absorption, steady-state, and time-resolved fluorescence spectroscopy. When PEG and 1-but4HTMSA are added to aqueous COU solutions, they result in a significant enhancement of fluorescence intensity, accompanied by detectable changes in emission maxima and variations in excited-state lifetimes, indicating pronounced nanoenvironmental modulation. Furthermore, iodide-ion-based fluorescence quenching experiments are carried out to investigate COU accessibility within the PEG/1-but4HTMSA system.

## 2 Experimental Section

### 2.1 Materials

Chemicals including 1,2,4-triazole, 1-bromobutane, tetrahydrofuran, 1,8-diazabicyclo[5.4.0]­undec-7-ene, and methanesulfonic acid were procured from AVRA (India). Coumarin, poly ethylene glycol 20 000 (molecular weight ≈ 20 000 g/mol), and potassium iodide were obtained from SRL (India) and used without further purification. Details regarding the purity of all chemicals are provided in the Supporting Information (Table S2). All glassware was thoroughly washed with distilled water, rinsed with acetone, and dried in a hot-air oven at 100 °C before use.

### 2.2 Synthesis of 1-Butyl-1,2,4-Triazolium Methanesulfonate (1-but4HTMSA)

Following established procedures, IL, 1-but4HTMSA, was synthesized as described in Scheme 1 (Supporting Information).^26^ The resulting 1-but4HTMSA is readily soluble in water, methanol, ethanol, acetonitrile, chloroform, and ethyl acetate, confirming its hydrophilic IL nature. ^1^H and ^13^C NMR spectra were recorded in CDCl_3_ on a Bruker AVANCE 400 FT-NMR spectrometer to confirm the structure and purity of 1-but4HTMSA.

#### 2.2.1 Spectral Data of 1-but4HTMSA

^1^H NMR (CDCl_3_, 400 MHz): 10.03 (s, 1H, 3CH), 8.89 (s, NH), 8.76 (s, 1H, 5CH), 4.47 (t, 2H, C*H*_2_–CH_2_–CH_2_–CH_3_), 2.79 (s, 3H, C*H*_3_SO_3_^–^), 1.94 (m, 2H, CH_2_–C*H*_2_-CH_2_–CH_3_), 1.36 (m, 2H, CH_2_–CH_2_–C*H*_2_–CH_3_), 0.95 (t, 3H, CH_2_–CH_2_–CH_2_–C*H*_3_). ^13^C NMR (CDCl_3_, 100 MHz): 143.53 (C-3), 141.52 (C-5), 51.61 (*C*H_2_–CH_2_–CH_2_–CH_3_), 39.43 (*C*H_3_SO_3_^–^), 30.79 (CH_2_–*C*H_2_–CH_2_–CH_3_), 19.25 (CH_2_–CH_2_–*C*H_2_–CH_3_), 13.26 (CH_2_–CH_2_–CH_2_–*C*H_3_).

### 2.3 Instrumentation and Methods

#### 2.3.1 Absorption and Steady-State Fluorescence Experiments

UV–visible absorption spectra (200–800 nm) were recorded on a Shimadzu UV-1900 double-beam spectrophotometer. Fluorescence measurements were performed using an Agilent Technologies Cary Eclipse fluorescence spectrometer with the excitation wavelength for COU set at 275 nm.

#### 2.3.2 Fluorescence Lifetime Measurement Experiments

Excited-state lifetime measurements of COU in various PEG+IL mixtures were carried out using a FLUOROLOG FL3-11 time-correlated single-photon counting system equipped with a nano-LED excitation source (N-LED 295 nm). Fluorescence decay curves were analyzed with DAS6 software (Horiba Jobin Yvon Ltd.) using a multiexponential fitting equation.^39^

1 $$I\left(t\right)=\Sigma \alpha_{i}e^{-\left(t/\tau_{i}\right)}$$

In this expression, α_i_ denotes the amplitude corresponding to the *i*th lifetime with the *i*th lifetime τ*_i_*. The average lifetime ⟨τ⟩ of COU samples was determined using the following equation.^39^

2 $$\left\langle \tau \right\rangle =\sum \alpha_{i}\tau_{i}/\sum \alpha_{i}$$

#### 2.3.3 Sample Preparation for Fluorescence Studies

An appropriate amount of COU was dissolved in ethanol. The final concentration of COU was fixed as 10 μM. The stock solutions of both PEG (30%) and 1-but4HTMSA (100 mM) were prepared in water. Various concentrations of PEG (3, 6, 9, 12, 15, 17, 21, 24, 27, 30%) and 1-but4HTMSA (100, 200, 300 μM) were then prepared by serial dilution of the respective stock solutions with Milli-Q water to samples containing COU (10 μM). Quenching measurements were performed on different mixtures of COU, PEG, and 1-but4HTMSA containing increasing amounts of KI (0–1 M). All samples were allowed to equilibrate for 2 h prior to measurement. For all fluorescence experiments, a small known volume of this stock solution was subsequently diluted with water, resulting in a final ethanol content of 1% (v/v) in all samples. Figure 1 schematically illustrates the overall COU/1-but4HTMSA/PEG system investigated in this study.

**Figure 1:**
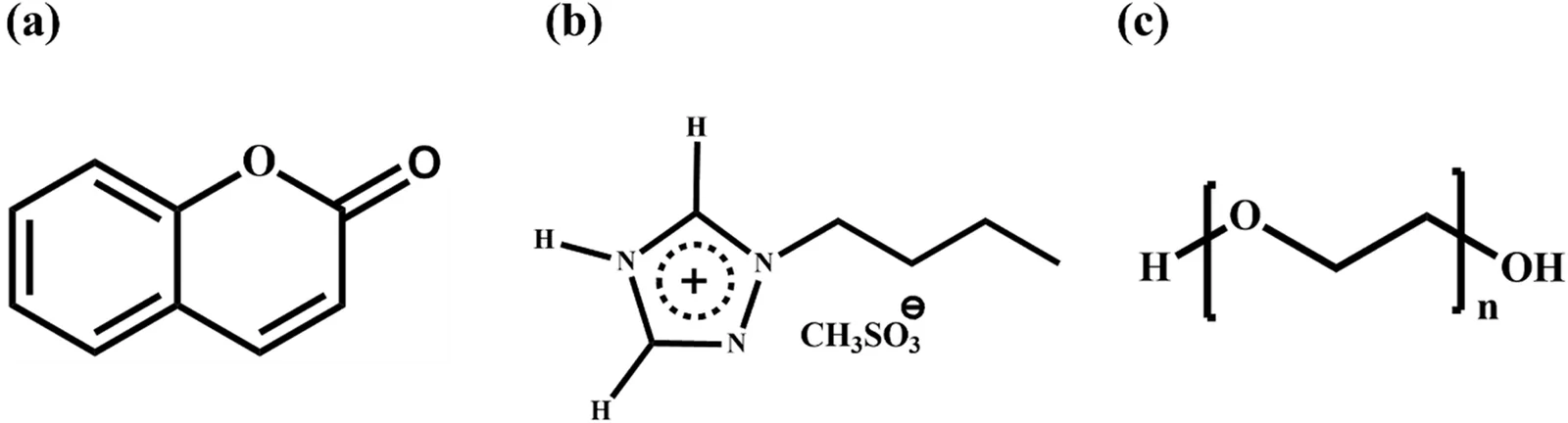
Chemical structure of (a) coumarin (COU), (b) 1-butyl-1,2,4-triazolium methanesulfonate (1-but4HTMSA), and (c) polyethylene glycol (PEG).

## 3 Results and Discussion

### 3.1 Absorption and Fluorescence Studies of COU in PEG/1-but4HTMSA Media

#### 3.1.1 Photophysical Behavior of COU in 1-but4HTMSA Media

In this study, the steady-state photophysical behavior of COU (10 μM) was investigated in aqueous solutions containing varying concentrations (0–300 μM) of 1-but4HTMSA. Representative UV–visible absorption and fluorescence emission spectra are shown in Figure 2.

**Figure 2:**
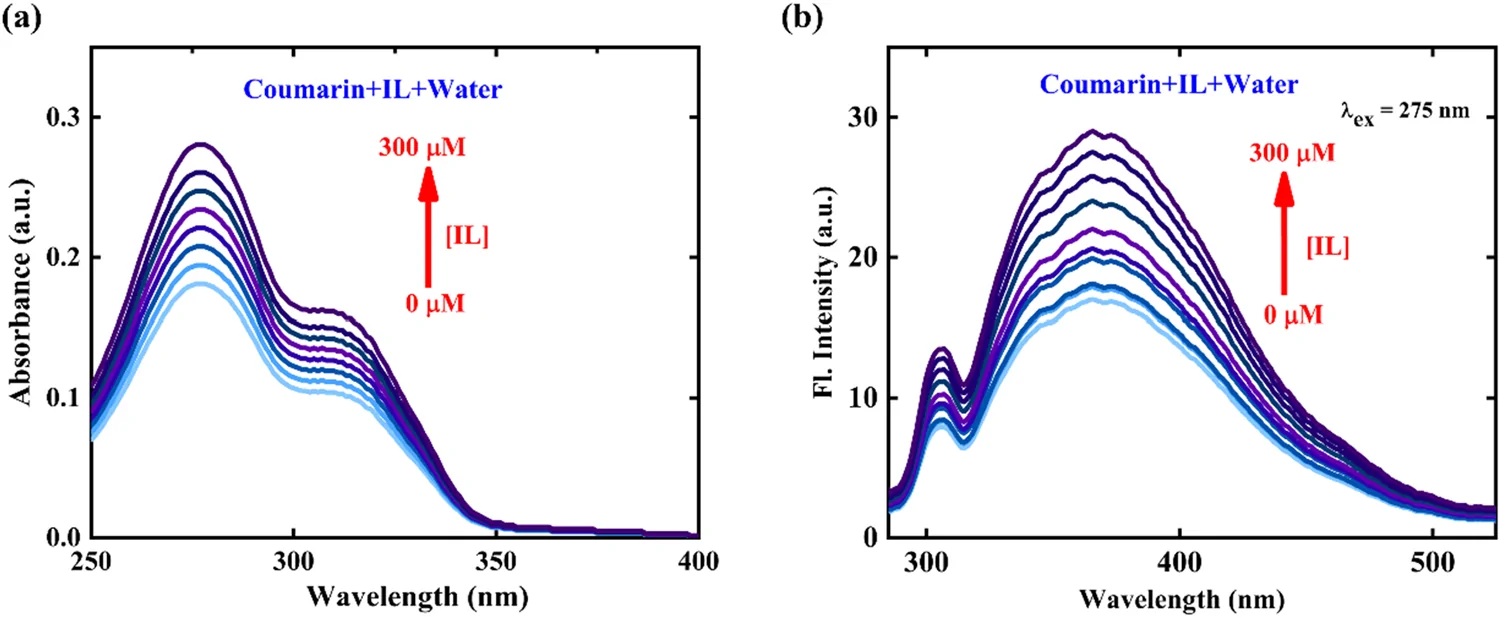
(a) UV–vis absorption and (b) fluorescence spectra of COU (λ_ex_ = 275 nm; 10 μM) with different concentrations of IL (0 mM–0.3 mM).

The absorption spectra of COU in water and aqueous 1-but4HTMSA solutions exhibit two characteristic UV bands at approximately 275 and 310 nm, which are attributed to π–π* electronic transitions of the COU chromophore.^40^ Notably, no significant shift in the absorption maximum is observed upon increasing 1-but4HTMSA concentration in the range of 0–300 μM, indicating that the electronic structure of the ground state of COU molecules remains largely unperturbed. However, absorbance exhibits a gradual enhancement with 1-but4HTMSA concentration, which can be attributed to enhanced solubilization of COU and changes in the local environment provided by the 1-but4HTMSA+water system. Figure 2b presents the fluorescence emission spectra of COU recorded upon excitation at 275 nm. A small enhancement in fluorescence intensity is observed around 380 nm with increasing 1-but4HTMSA concentration.

#### 3.1.2 PEG-Induced Modulation of COU Fluorescence

To systematically elucidate the influence of the microenvironment on the photophysical behavior of COU, fluorescence studies were first carried out in aqueous 1-but4HTMSA solutions. Subsequently, COU was investigated in PEG systems to examine the role of polymer-induced microenvironmental effects. Increasing PEG concentration progressively alters the local polarity, viscosity, and structural organization around the fluorophore,^41,42^ enabling the assessment of how PEG confinement and restricted solvent relaxation alone influence the spectral position, intensity, and excited state of COU. COU fluorescence spectra in varying concentrations of PEG media shown in Figure 3 display a single broad emission band without vibrational structure. The observed enhancement in fluorescence intensity is attributed to the restricted rotational motion of COU in the higher hydrophobic medium, which suppresses nonradiative deactivation pathways from the S_1_ excited state. A pronounced blue shift (≈40 nm) in emission maxima represents higher hydrophobicity of the system in PEG (Figure 3b). PEG improves COU solubilization and prevents aggregation-induced quenching.

**Figure 3:**
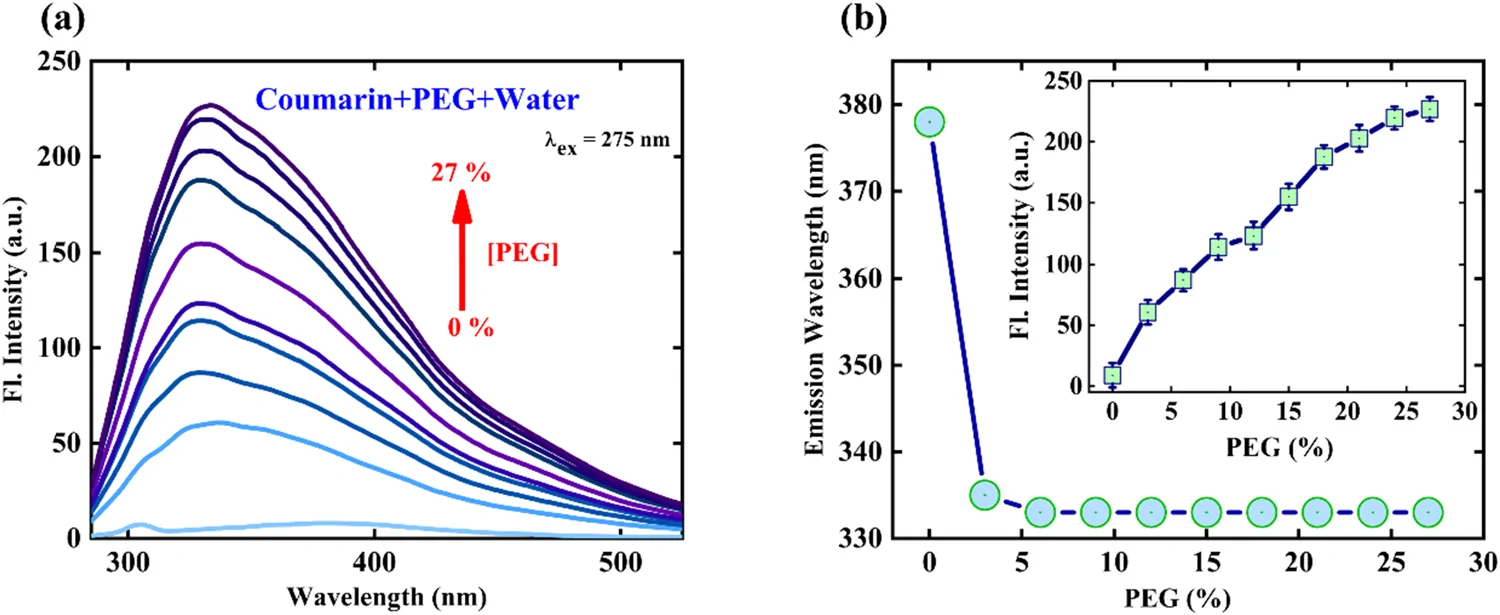
(a) Fluorescence spectra of COU (λ_ex_ = 275 nm; 10 μM) with different concentrations of PEG. (b) Variation of fluorescence parameters of COU with different concentrations of PEG [3–27%]. (b) Emission wavelength, (b-inset) fluorescence intensity.

#### 3.1.3 Effect of 1-but4HTMSA on COU in the PEG System

The synergistic effect of IL on COU incorporated into PEG matrices was explored by COU photophysics. The approach of evaluating modulation in fluorescence properties of COU (10 μM) allows the combined effect of PEG (3–27%) and 1-but4HTMSA (300 μM) on their cooperative contributions toward molecular association and polarity, which promotes confinement of COU within the polymer/1-but4HTMSA hybrid systems. Here, we have added specific concentrations of 1-but4HTMSA to the preformed COU/PEG system.

The COU fluorescence in the PEG/1-but4HTMSA system is illustrated in Figure 4. In the presence of 1-but4HTMSA, the overall fluorescence intensity increases as compared to that of PEG alone, with a slight shift in the emission peak; notably, even at low PEG concentrations, 1-but4HTMSA enhances emission, indicating its influence even in the dilute regime. The addition of 1-but4HTMSA to the COU/PEG system results in an enhancement of fluorescence intensities, indicating strengthened microenvironmental stabilization and improved excited-state behavior of COU without introducing aggregation. This confirms that 1-but4HTMSA synergistically enhances the COU/PEG photophysical response (Figure 4b).

**Figure 4:**
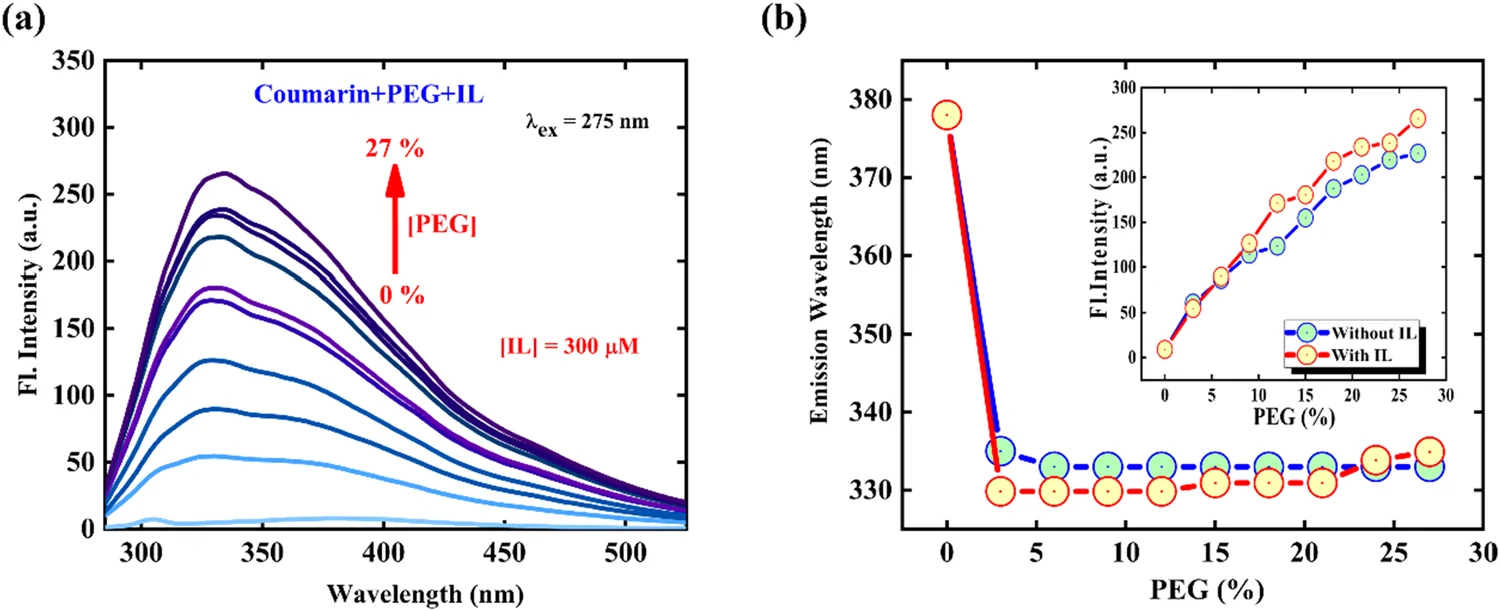
(a) Fluorescence spectra of COU (λ_ex_ = 275 nm; 10 μM) with different concentrations of PEG in the presence of 1-but4HTMSA (300 μM). (b) Variation of fluorescence parameters of COU with different concentrations of the PEG/1-but4HTMSA system. (b) Emission wavelength, (b-inset) fluorescence intensity.

### 3.2 Fluorescence Lifetime Studies

To gain information on the molecular interaction between PEG and 1-but4HTMSA, COU fluorescence lifetime decay measurements were performed at different concentrations of PEG and 1-but4HTMSA. The resulting lifetime parameters offer valuable information on the excited-state dynamics of COU in different heterogeneous media. Fluorescence decay of COU in water is biexponential with a 0.4 ns lifetime (τ). This could be due to a very fast nonradiative decay in water, strong solvation-induced quenching, high polarity, and H-bonding of water, which destabilizes the excited state.^40^ Decay of COU in the presence of 1-but4HTMSA and PEG is well fitted by a triexponential model with χ^2^-value as 1.07. The average lifetime of COU can arise from the varying relative populations of its three species in the excited state. The corresponding deconvoluted lifetime parameters are presented in Table S2. Adding 1-but4HTMSA (100–300 μM) to an aqueous solution of COU slightly decreases τ to 0.13 ns. This indicates mild restriction of nonradiative pathways and weak interaction between 1-but4HTMSA and COU. PEG dramatically enhances the lifetime (3.38 ns) by approximately 8.5-fold from water. PEG provides protective hydrophobic pockets for COU. The fluorescence lifetime saturates at around 15–27% PEG, indicating that COU molecules are fully embedded, and further increases in PEG concentration do not alter the local rigidity. Upon addition of 1-but4HTMSA (100–300 μM) to the COU/PEG (15%) system, a slight decrease in lifetime of 2.59 ns is observed. 1-but4HTMSA may compete with COU and slightly disturb the polymer microenvironment. But the system remains largely rigid and confined (Figure 5).

**Figure 5:**
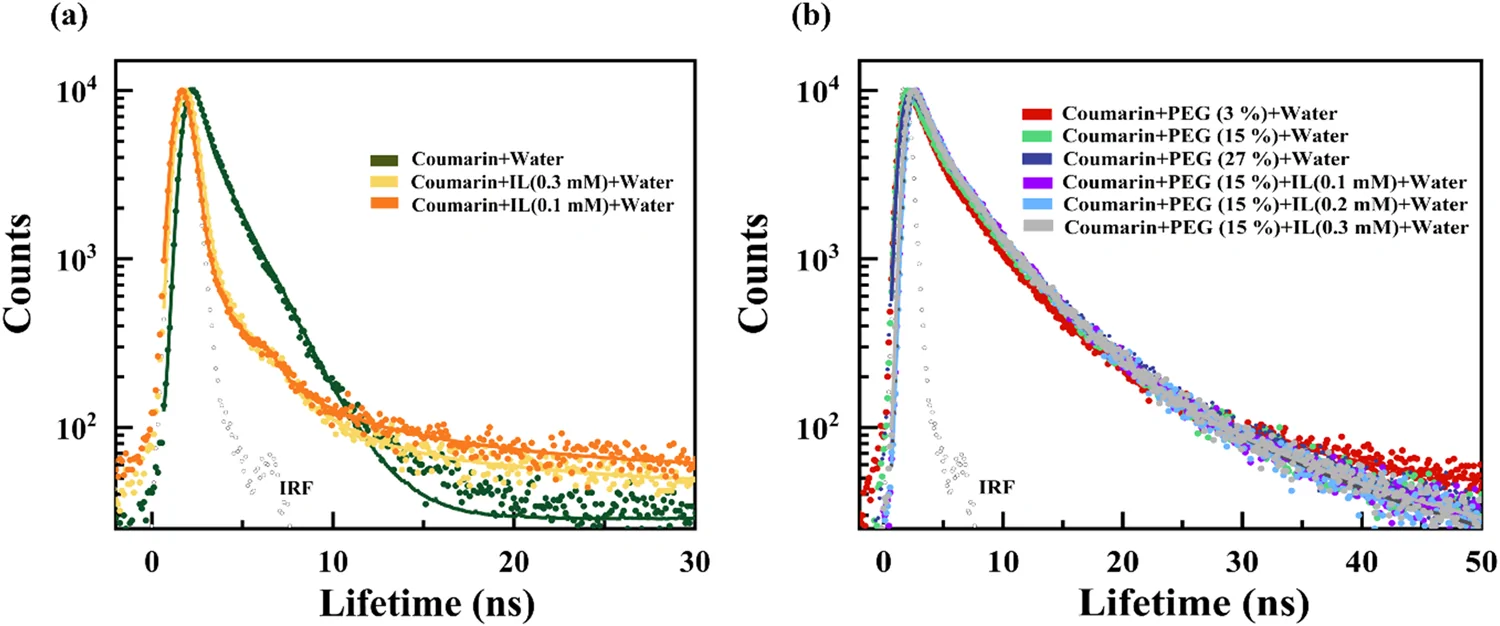
(a) and (b) Fluorescence lifetime decay profiles of COU (λ_ex_ = 275 nm; 10 μM) at varying concentrations of 1-but4HTMSA and PEG.

### 3.3 Quenching Studies

Furthermore, a quenching study was performed to investigate the accessibility and local microenvironment of the COU. This study allowed discrimination between static and dynamic quenching mechanisms and helped confirm the effective shielding and stabilization of COU within the PEG/1-but4HTMSA domains. In this study, potassium iodide (KI) was used as a quencher.

KI efficiently interacts with the excited state of COU, resulting in dynamic quenching that occurs exclusively during the excited state lifetime. A clear linear dependence can be seen in the Stern–Volmer plot of *F*_0_/*F* versus [KI], suggesting that the quenching process adheres to the classical Stern–Volmer relationship and that no substantial ground-state COU-KI complex formation takes place. This demonstrates the complete dynamic nature of observed quenching. The substantial amount of quenching suggests that COU is not completely enclosed by highly protected hydrophobic domains. Rather, iodide ions can still reach the fluorophore because of the PEG/1-but4HTMSA matrix’s remaining openness. On the basis of the slope of the linear fit, a small intercept of 1.09382, and a strong correlation value (*R* ^2^ = 0.967), the Stern–Volmer quenching constant (*K*_SV_), which is 0.50894, confirms excellent linearity and dependability of the quenching behavior (Figure 6).

**Figure 6:**
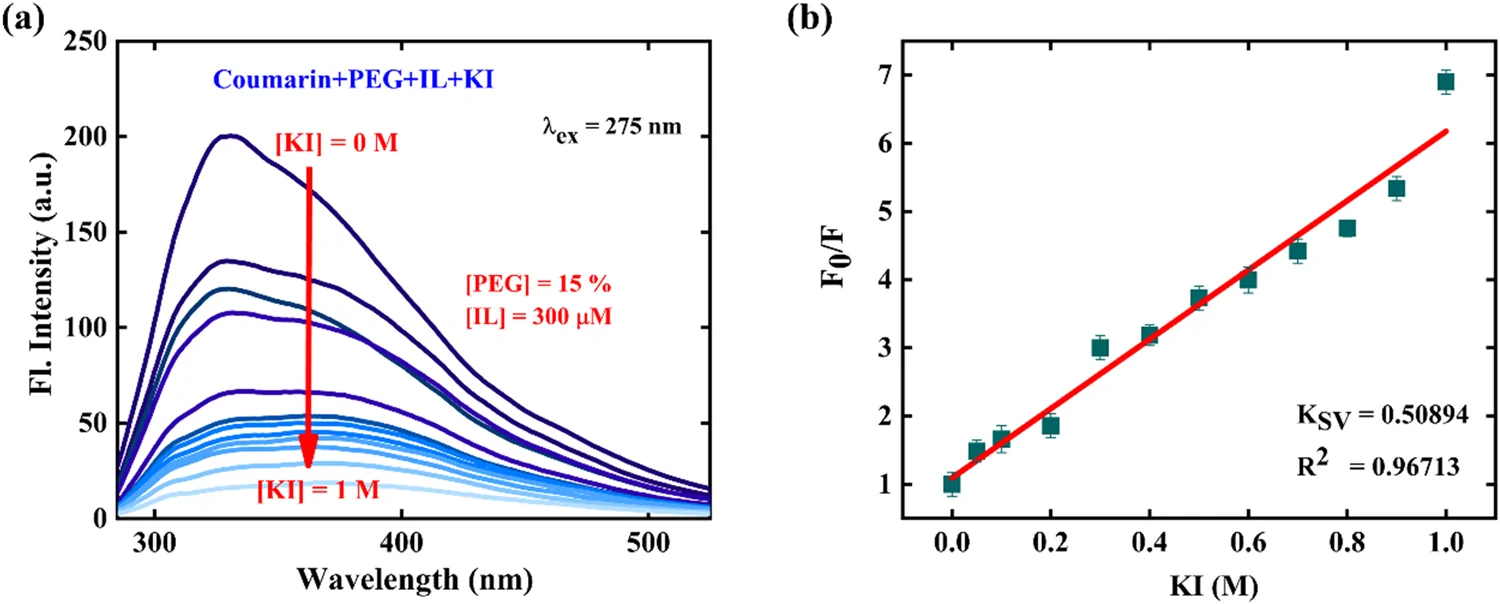
(a) KI-induced fluorescence quenching of COU (λ_ex_ = 275 nm; 10 μM) in the PEG/1-but4HTMSA system. (b) Stern–Volmer plot of $\frac{F_{0}}{F}$ versus [KI].

## 4 Discussion

COUs are extremely sensitive to their surroundings, especially to variations in polarity, microviscosity of medium, and certain solute–solvent interactions.^43–45^ 1-but4HTMSA provides a polar microenvironment conducive to efficient fluorescence emission, highlighting its potential employment as a tunable medium for photophysical properties. Both experimental investigations and theoretical calculations reveal that the parent COU possesses a lowest singlet excited state (S_1_) of n to π* character, which accounts for its inherently weak fluorescence.^43^ The reduction of nonradiative decay routes is indicated by the alteration of COU fluorescence in the presence of 1-but4HTMSA. This is probably due to the limited molecular motion of COU molecules and advantageous electrostatic and hydrogen-bonding interactions with 1-but4HTMSA. Moreover, the presence of the 1-but4HTMSA may result in a more ordered arrangement surrounding the fluorophore, stabilizing the excited state.

According to reports, coupling of triazole-linked COUs with PEG creates a tunable microenvironment that modulates fluorescence behavior through hydrophilic–hydrophobic balance, solvation effects, and supramolecular interactions.^46^ Systematic studies have demonstrated that polymer matrices significantly influence the fluorescence decay of dialkylamino-substituted COUs and the photophysical behavior of photoresponsive hyperbranched polyesters.^45,47^ Furthermore, COUs have been employed as structural motifs in fluorescence probes based on intramolecular quenching and as reporters of radical processes in solutions and thin polymer films;^44,48^ notably, Donovalová et al. investigated the spectral characteristics of COU derivatives in both solution and polymer matrix environments.^49^ PEG creates a heterogeneous microenvironment that can significantly influence the aggregation state and fluorescence response of embedded fluorophores. Recent studies further indicate that PEG enhances the solubilization of hydrophobic dyes in water by forming hydrogen-bonded domains and flexible chain conformations that stabilize dispersed molecules,^42^ although elevated polymer concentrations may induce aggregation due to π–π stacking of dyes and crowding effects.^50^

In the present system, COU was physically associated through hydrophobic–hydrophilic balance with the PEG without any chemical conjugation. The gradual increase in PEG concentration leads to a less polar and more structured microenvironment around the COU molecules compared to pure water. This reduced polarity preferentially destabilizes the excited π–π* state relative to the ground state, resulting in emission at shorter wavelengths. In addition, increased restricted solvent relaxation in the PEG-rich medium limits the stabilization of the excited state, further contributing to the observed blue shift in fluorescence emission. Spectral analysis of COU in PEG and PEG/1-but4HTMSA systems revealed that the emission wavelength remained nearly constant across increasing PEG concentrations, indicating minimal shifts in its photophysical environment. However, the fluorescence intensity gradually increased with PEG content and was consistently higher in the presence of 1-but4HTMSA.

PEG modulates the excited states of COU and their relaxation pathways through a combination of specific and nonspecific interactions, such as dipole–dipole interactions and hydrophobic interactions, which can stabilize the excited states. The solvation of COU in the PEG is impacted by the behavior of 1-but4HTMSA and its aqueous environment, which has a distinct and limited mobility. This examines whether the solvation environment of COU undergoes reorganization within the PEG matrix during its excited-state lifetime upon the incorporation of 1-but4HTMSA, and how such reorganization impacts the emission properties of COU. As reported in the classical study by de Melo et al., the n-to-π* excited state shows strong sensitivity to solvent polarity and predominantly favors nonradiative decay, while π-to-π* state is intrinsically more emissive and is stabilized in less polar and more rigid environments.^43^ In aqueous media, the high polarity stabilizes the n-to-π* excited state of COU, resulting in a shorter fluorescence lifetime, as reflected by the faster decay component in the TCSPC profiles.^43^ A more viscous and structurally restricted nanoenvironment is created in PEG-containing systems as the polymer is gradually added, which restricts solvent relaxation and intramolecular movements. Such conditions preferentially stabilize the π-to-π* state, suppress nonradiative decay, and produce systematically longer fluorescence lifetimes, in line with the observed decay kinetics. The incorporation of 1-but4HTMSA into the PEG further fine-tunes the local polarity and rigidity, reinforcing lifetime changes without causing a significant shift in the emission maximum, consistent with steady-state observations.

The alteration of the photophysical properties arises from the medium-induced suppression of COU molecular rotation in the PEG/1-but4HTMSA system. The stiffening of the PEG/1-but4HTMSA matrix, which results from dipole–dipole interactions between PEG and 1-but4HTMSA within the hydrophobic confinement of PEG, is responsible for this impact. Although PEG provides the primary confined microenvironment, 1-but4HTMSA plays a critical role as a microenvironmental modulator. The modest decrease in lifetime upon addition indicates that 1-but4HTMSA partially reorganizes the polymer network, enabling the controlled adjustment of dye exposure and accessibility rather than complete encapsulation. The quenching results strongly support this interpretation. The linear Stern–Volmer behavior and appreciable quenching constant indicate dynamic quenching by iodide ions, confirming that COU remains accessible to external quenchers even within the PEG/1-but4HTMSA domains. This accessibility is consistent with the lifetime trends and rules out excessive shielding or the formation of rigid, impermeable pockets. Instead, the PEG/1-but4HTMSA matrix provides a balanced environment that stabilizes the emissive state of COU while maintaining controlled openness, in agreement with steady-state and time-resolved observations. This tunability of 1-but4HTMSA is essential for designing responsive and selective fluorescence-sensing platforms, where the additive can serve as a functional regulator that optimizes sensitivity, interaction strength, and analyte recognition.

## 5 Conclusion

This study establishes the central role of polymers in designing advanced pharmaceutical excipient systems by demonstrating that the PEG/1-but4HTMSA framework effectively modulates the nanoenvironment of hydrophobic COU in aqueous media. To the best of our knowledge, this is the first study to demonstrate the use of a triazolium PIL in combination with PEG as an approach for enhancing the solubilization and photophysical properties of coumarin in aqueous media, where the cooperative interaction between PEG and the PIL alters the local polarity and photophysical behavior. The incorporation of a 1,2,4-triazolium-based PIL enables enhanced solubility, stability, and controlled molecular organization through favorable intermolecular interactions. Quenching studies with potassium iodide further confirm dynamic accessibility of COU, as evidenced by the linear Stern–Volmer analysis. Overall, the integration of steady-state, lifetime, and quenching measurements establishes that 1-but4HTMSA incorporation allows precise modulation of local polarity, structural organization, and accessibility without compromising fluorescence efficiency. These results highlight the benefit of PEG/1-but4HTMSA systems as flexible and robust platforms for the creation of sensing materials based on responsive fluorescence as well as functional polymer-supported photonic and biomedical applications.

## Supplementary Material



## Notes

Table S1: chemical information; Scheme S1: synthesis of IL; Figures S1 and S2: NMR spectra; Figure S3: absorbance and fluorescence intensity plots; Table S2: fluorescence lifetime data (PDF)
