# Supplementary material for: Photophysical Investigation of Coumarin Solubilization in Polyethylene Glycol Mediated by a 1,2,4-Triazolium Protic Ionic Liquid
Source: ACS Omega. 2026 Aug 14;11(33):50334–41. doi: 10.1021/acsomega.6c06167 (PMC13520030; doi:10.1021/acsomega.6c06167)
Supplement: Supplementary file 1 [file ao6c06167_si_001.pdf]

Photophysical Investigation of Coumarin Solubilization in Polyethylene Glycol Mediated by a 1,2,4-Triazolium Protic Ionic Liquid

Saranya Cheriyathennatt[a,b], *Surya Saravanan*[c], *Srinivasan Gokul Raj*[c], *Susithra Selvam*[d]*, *Elango Kandasamy*[a,b]*

[a]Department of Chemistry, Amrita School of Physical Sciences, Coimbatore, Amrita Vishwa Vidyapeetham, India

[b]Functional Materials Laboratory, Amrita School of Engineering, Coimbatore, Amrita Vishwa Vidyapeetham, India

[c]Department of Physics, Pondicherry University, Kalapet, Puducherry, 605014, India

[d]Department of Chemistry, Ethiraj College For Women, Ethiraj Salai, Egmore, Chennai, Tamil Nadu 600008, India

Supporting Information

Table S1 Purity of chemicals used

S. No.	Chemical	Source	Purity
1	1,2,4-triazole	Avra Laboratories Pvt. Ltd, India	98%
2	Tetrahydrofuran	Avra Laboratories Pvt. Ltd, India	99%
3	1,8-Diazabicyclo[5.4.0]undec-7-ene	Avra Laboratories Pvt. Ltd, India	97%
4	1-bromobutane	Avra Laboratories Pvt. Ltd, India	98%
5	Methanesulfonic acid	Avra Laboratories Pvt. Ltd, India	98%

7	Coumarin	Sisco Research Laboratories Pvt. Ltd. (SRL), India	Pure, assay 98%

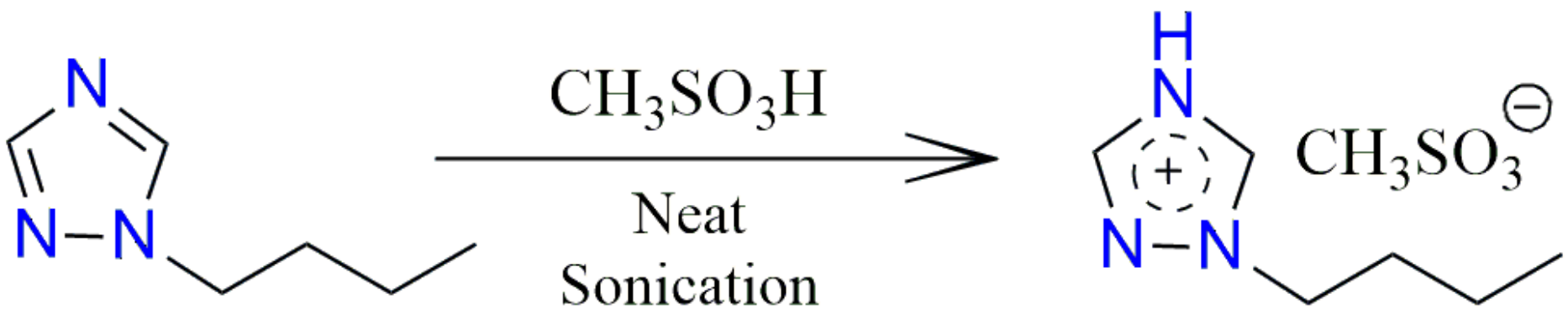

Scheme S1 Synthesis of 1-butyl-1,2,4-triazolium methanesulfonate (1-but4HTMSA)

NMR Spectra of 1-but4HTMSA

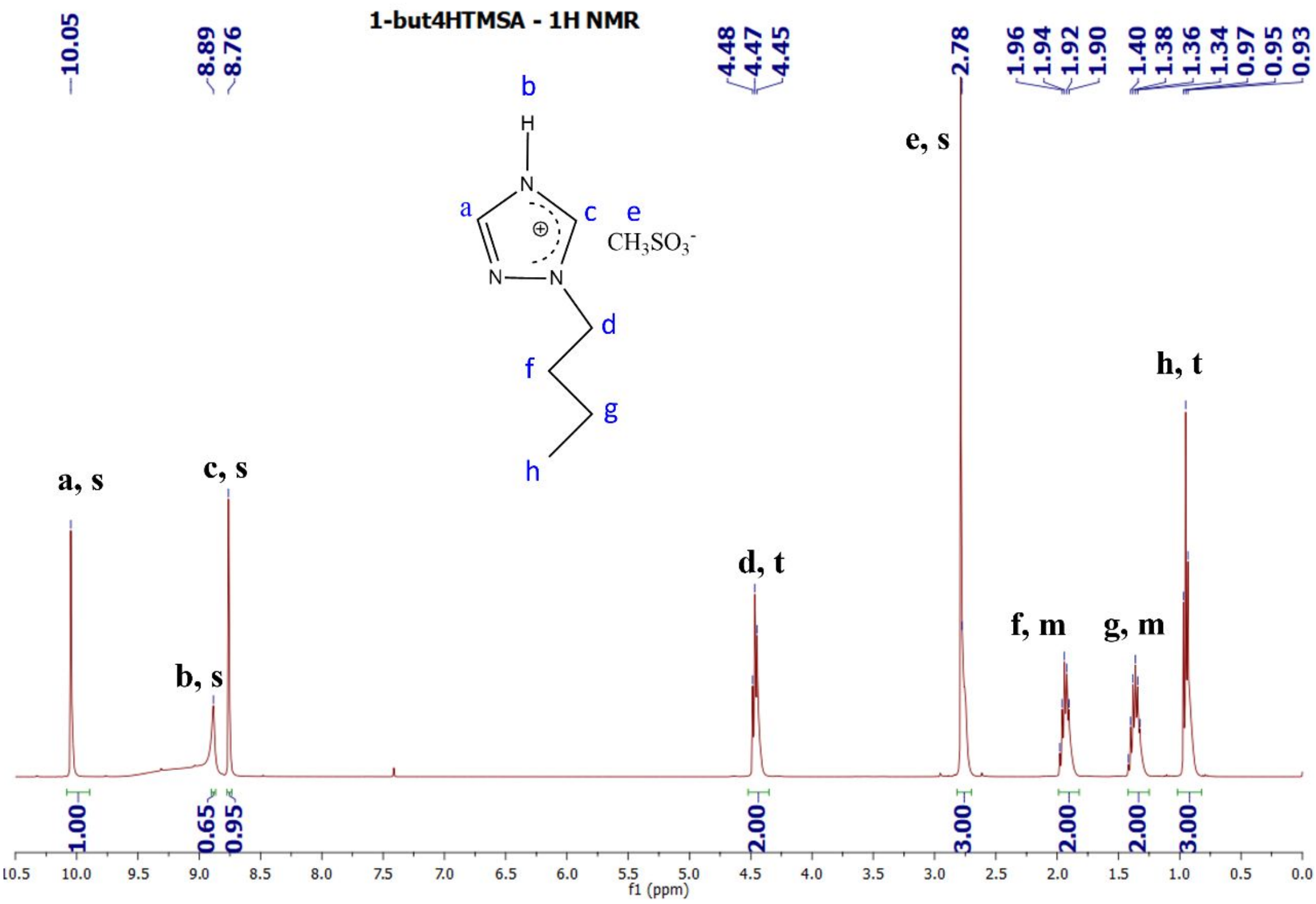

Fig. S1 ^{1}H NMR spectra of 1-but4HTMSA

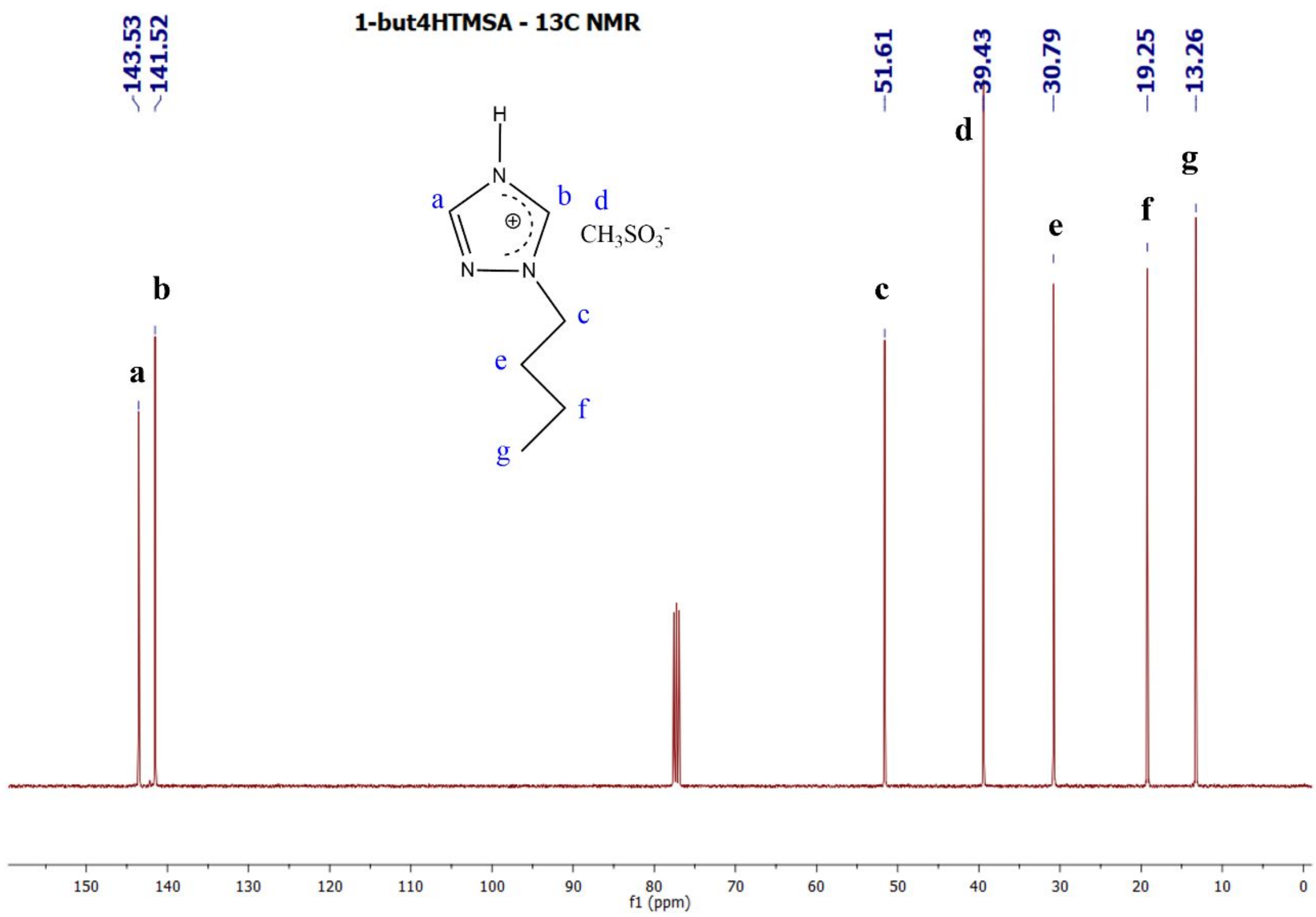

Fig. S2 ^{13}C NMR spectra of 1-but4HTMSA

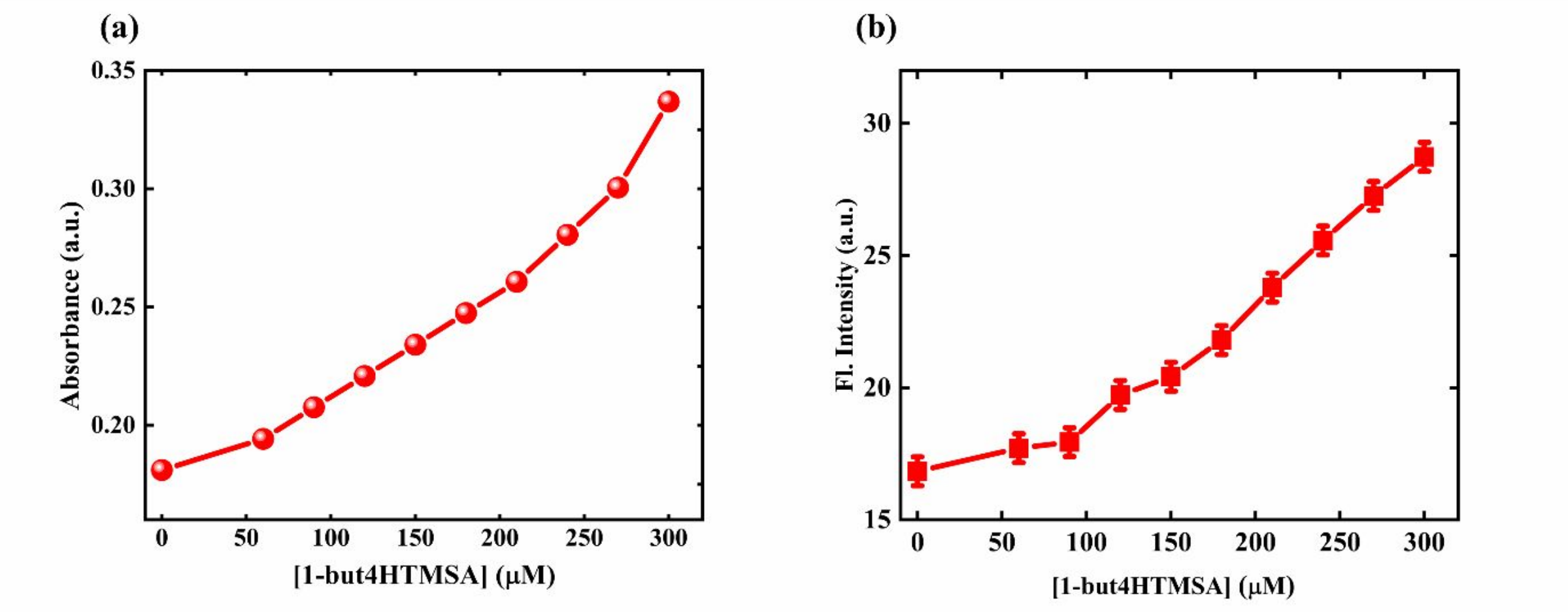

Fig. S3 Variation of absorbance and fluorescence intensity of COU with different concentrations of IL

TCSPC Measurements

Table S2 Fluorescence lifetime parameters

System	τ1 (ns)	τ2 (ns)	τ3 (ns)	α1	α2	α3	τ_{avg}	χ2
COU (10 μM) + Water	1.88	0.04	---	0.23	0.76	---	0.4	1.99
COU (10 μM) + IL (100 μM) +Water	11.29	2.28	0.20	0.001	0.01	0.98	0.23	1.25
COU (10 μM) + IL (300 μM) +Water	0.11	1.87	9.01	0.1	0.06	0.007	0.13	1.07
COU (10 μM) + PEG (3 %) +Water	4.09	13.62	1.18	0.36	0.02	0.61	2.54	1.06
COU (10 μM) + PEG (15 %) +Water	4.34	1.14	15.24	0.47	0.69	0.03	3.33	1.09
COU (10 μM) + PEG (27 %) +Water	2.032	5.37	18.07	0.67	0.30	0.02	3.39	1.92
COU (10 μM) + PEG (15 %) + IL (100 μM) +Water	4.24	14.46	1.10	0.38	0.02	0.58	2.67	1.06
COU (10 μM) + PEG (15 %) + IL (200 μM) +Water	3.97	13.02	0.95	0.39	0.03	0.57	2.65	1.10
COU (10 μM) + PEG (15 %) + IL (300 μM) +Water	4.35	15.80	1.10	0.37	0.02	0.60	2.59	1.12
